# A Single Nucleotide C3 Polymorphism Associates With Clinical Outcome After Lung Transplantation

**DOI:** 10.3389/fimmu.2019.02245

**Published:** 2019-09-26

**Authors:** Tineke Kardol-Hoefnagel, Kevin Budding, Eduard A. van de Graaf, Jessica van Setten, Oliver A. van Rossum, Erik-Jan D. Oudijk, Henderikus G. Otten

**Affiliations:** ^1^Center for Translational Immunology, University Medical Center Utrecht, Utrecht, Netherlands; ^2^Department of Respiratory Medicine, University Medical Center Utrecht, Utrecht, Netherlands; ^3^Department of Cardiology, University Medical Center Utrecht, Utrecht, Netherlands; ^4^Center of Interstitial Lung Diseases, St. Antonius Hospital, Nieuwegein, Netherlands

**Keywords:** lung transplantation, complement component 3, acute rejection, bronchiolitis obliterans syndrome, BALF, transplantation genetics

## Abstract

**Background:** Development of chronic rejection is still a severe problem and causes high mortality rates after lung transplantation (LTx). Complement activation is important in the development of acute rejection (AR) and bronchiolitis obliterans syndrome, with C3 as a key complement factor.

**Methods:** We investigated a single nucleotide polymorphism (SNP) in the C3 gene (rs2230199) in relation to long-term outcome after LTx in 144 patient-donor pairs. In addition, we looked at local production of donor C3 by analyzing bronchoalveolar lavage fluid (BALF) of 6 LTx patients using isoelectric focusing (IEF).

**Results:** We demonstrated the presence of C3 in BALF and showed that this is produced by the donor lung based on the genotype of SNP rs2230199. We also analyzed donor and patient SNP configurations and observed a significant association between the SNP configuration in patients and episodes of AR during 4-years follow-up. Survival analysis showed a lower AR-free survival in homozygous C3 slow patients (*p* = 0.005). Furthermore, we found a significant association between the SNP configuration in donors and BOS development. Patients receiving a graft from a donor with at least one C3 fast variant for rs2230199 had an inferior BOS-free survival (*p* = 0.044).

**Conclusions:** In conclusion, our data indicate local C3 production by donor lung cells. In addition, a single C3 SNP present in recipients affects short-term outcome after LTx, while this SNP in donors has an opposite effect on long-term outcome after LTx. These results could contribute to an improved risk stratification after transplantation.

## Introduction

Lung transplantation (LTx) is the final treatment option for patients with end-stage lung diseases. Despite improved immunosuppressive drugs and short-term survival, 28% of recipients experience at least one episode of acute rejection (AR) during the 1st year after LTx (1). Among other causes, AR may lead to the development of pulmonary dysfunction with chronic lung allograft dysfunction (CLAD) as the most common final outcome. CLAD is predominantly a result of chronic rejection, presenting as restrictive (restrictive allograft syndrome, RAS), or as obstructive (bronchiolitis obliterans syndrome, BOS), the most observed form (2). Mainly due to the high incidence of CLAD, long-term survival rates for those patients remain low. Chronic rejection can be mediated by both cellular and humoral immunity (3) that could initiate the complement system.

In solid organ transplantation, complement proteins play an important role in both acute, and chronic rejection (4). In a prospective cohort study it was shown that increased levels of complement proteins C4a and C5a early after LTx were associated with primary graft dysfunction (5). Regarding antibody-mediated acute rejection, it has been suggested that complement activation by binding of C1q to donor specific antibodies, either preformed, or *de novo*, play a role, although the mechanism is not fully elucidated. It has been shown that patients with human leukocyte antigen antibodies have increased C4d deposition in lung biopsies (6), and in antibody-mediated acute rejection, higher C4d levels in bronchoalveolar lavage fluid (BALF) were found (7). Concerning their role in chronic rejection, it is known that lung biopsies of patients who developed chronic graft dysfunction showed an increased C3d deposition (8). Also, during BOS complement regulatory proteins are downregulated while factors indicating complement activation like C3a are increased in BALF (9).

A key player in the complement cascade is complement C3, which is involved in all three pathways. Although serum complement factors, including C3, are mainly produced by hepatocytes, tissue cells and also human alveolar macrophages and type II epithelial cells are able to produce, and secrete complement factors locally (10, 11).

The coding region of the beta chain of complement C3 exhibits a single nucleotide polymorphism (SNP) rs2230199 that results in an amino acid change at position 102 [Arg(+)>Gly(–)], thereby switching the isoelectric point (pI) from 5.86 to 5.81 (12). The resulting two C3 variants C3 slow (C3S) and C3 fast (C3F) are common allotypes named after their different electrophoretic motility. The allelic frequency of C3F is 20% in Caucasian populations.

This SNP is associated with complement-dependent disease phenotypes like age-related macular degeneration (13) and is also studied in kidney and liver transplantation. Some studies show an association with graft loss, but results are conflicting, and could not be reproduced in larger cohorts (14–19). No data are currently available about the relation between this C3 polymorphism and outcome after LTx. To fill this gap and take into consideration the importance of complement proteins—and the pivotal role of C3—in transplant outcome, we studied the effect of SNP rs2230199 on both short- and long-term LTx outcome.

## Patients and Methods

### Patients

For this study, we included 144 patients and their respective donors that underwent a LTx between January 2004 and December 2013. All study participants gave informed consent and the study was approved by the medical ethical committee of our center (METC 06–144). All patients received standardized immunosuppressive therapy consisting of tacrolimus, prednisolone, and mofetil mycophenolate. Patients at high risk for cytomegalovirus (CMV) reactivation (defined as patients having CMV positive serology or patients receiving a graft from a CMV-positive donor) received treatment with valganciclovir for up to 6 months.

AR was defined as a spontaneous decline of lung function that was reversed after steroid pulse treatment and for which other causes of lung function decline were excluded. BOS was diagnosed according to international guidelines as the forced expiratory volume in 1 s that is ≤80% of baseline in absence of any other cause of disease (20).

Samples of donor blood and patient blood were collected prior to or during transplantation procedure. From each respective sample, peripheral blood mononuclear cells were isolated using Ficoll-paque gradient centrifugation and stored in liquid nitrogen until further analysis. LTx patients underwent a bronchoalveolar lavage upon suspicion of a rejection episode. BALF was collected and processed according to diagnostic protocol. Samples were frozen at −80 until usage.

### DNA Isolation and Genotyping

Frozen PBMC samples were thawed at 37°C and dissolved in RPMI-1640 (Life Technologies, California, USA) 20% fetal bovine serum (Bodinco, Alkmaar, The Netherlands), followed by 10 min centrifuging at 1,800 rpm. The obtained cell pellet was dissolved in phosphate buffered saline (Sigma, Missouri, USA) at a concentration of 10 × 10^6^ cells/ml. DNA was isolated with the MagnaPure Compact System (Roche Diagnostics, Basel, Switzerland), according to manufacturer's protocol.

One SNP, rs2230199, in the complement C3 gene that is frequently present in the Western European population was selected.

Samples were genotyped within the *i*GeneTRA*i*N network using the specifically designed and developed Affymetrix “TxArray,” which contains 767,203 variants (21, 22). Subsequently, to remove low-quality genotyped SNPs and samples analyses followed stringent quality control (QC) procedures. Samples with a missing rate >3% were removed. Furthermore, a subset of high-quality independent SNPs was generated according to the following conditions: missing rate <1%, Hardy-Weinberg *P* > 0.001, minor allele frequency >0.1, and LD pruning leaving no SNP-pairs with *r*^2^ > 0.2. Subsequently, samples were removed with heterozygosity >2 SD from the mean of all samples, related samples (keeping only one samples of each pair with proportion of IBD>0.2), and samples of non-European ancestry [based on principle component analysis using the 1,000 Genomes Project (Phase 1) populations as reference (23)].

SNPs were removed if they presented a missing rate >5%, Hardy-Weinberg *P* < 0.01, or when they were monomorphic. Untyped SNPs were imputed (24, 25) with the 1,000 Genomes Project (v3) (26), and the Genomes of The Netherlands (v5) (27) as reference panels. Samples were phrased with SHAPEIT (28), and imputed with IMPUTE v2 (29). Sequence data has been deposited at the European Genomephenome Archive (EGA), which is hosted by the EBI and the CRG, under accession number EGAS00001003843. Further information about EGA can be found on https://ega-archive.org “The European Genome-phenome Archive of human data consented for biomedical research” (http://www.nature.com/ng/journal/v47/n7/full/ng.3312.html).

### C3 SNP Analysis

One-time collected 5x diluted BALF was separated on a 4–20% kDa gel (Biorad, California, USA) followed by western blot analysis and staining with a primary biotin-linked sheep polyclonal anti-human C3 antibody (MyBiosource, California, USA) and a secondary SA-poly HRP antibody (Sanquin, Amsterdam, The Netherlands).

To analyze the C3 allotype in BALF of 6 LTx patients, proteins in lavage were acetone precipitated and resolved in rehydration buffer [8M Urea (GE-Healthcare Life Sciences, Hoevelaken, The Netherlands) 2% CHAPS (Sigma, Missouri, USA) 0.5% Bio-Lyte 5/7 Ampholytes (Biorad, USA) 0.002% Bromophenol Blue (Sigma, USA)]. 2 μg protein diluted in sample rehydration buffer was loaded per 7 cm ZOOM IPG strip pH 5.3–6.3 (Thermo Fisher, Massachusetts, USA). After overnight rehydration at room temperature, isoelectric focusing (IEF) was performed according to manufacturer's protocol using the Ettan IPGphor3 IEF system (GE Healthcare Life Sciences, The Netherlands). Briefly, an initial potential of 170 V was applied for 15 min, followed by a voltage ramp stage from 170 to 2,000 V for 45 min. Total protein was focused at 2,000 V for 105 min, max 50 μA per strip. Strips were equilibrated in 1x SDS-PAGE sample buffer for 15 min. Next, second dimension SDS-PAGE was performed on a 4–20% kDa protein gel (BioRad, USA) followed by western blotting. C3 was thereafter visualized with a HRP conjugated rabbit polyclonal antibody against recombinant human C3 (MyBiosource, USA).

### Statistical Analysis

We used SPSS version 25 (IBM Corp., Armonk, NY) and GraphPad Prism version 7.02 (GraphPad Software Inc., San Diego, CA) for statistical analyses. Categorical data were analyzed via the Fischer's exact test, and differences in continuous variables were assessed via ANOVA. Kaplan-Meier analyses were used for survival analyses and differences were analyzed via log-rank test and Cox regression with the following covariates: donor and recipient age, donor smoking stage, CMV, and EBV reactivation, transplantation year and AR episode. A *P* < 0.05 was considered to be statistically significant.

## Results

### Patient and Donor Characteristics

Table 1 describes patient and donor clinical and demographic characteristics according to the presence of the C3F allele. There were no significant differences between the both patient and donor genotype groups, except for the percentage non-heart beating donors (15.9 and 38.1% in SS and SF/FF donors, respectively).

**Table 1:** Clinical and demographic parameters of lung transplant patients and donors.

	**All**	**SS**	**FS/FF**	***p*-value**
**Patients**				
Total number	132	80	52	
**Gender**				
Male	61	35	26	0.300^a^
Female	71	45	26	
Mean age (years)	46 ± 13	47 ± 13	45 ± 14	0.318^c^
Mean follow-up (months)	77.3 ± 42	75.5 ± 44	80.1 ± 39	0.545^c^
**Primary disease**				
COPD	64	42	22	0.506^b^
CF	36	19	17	
ILD	31	18	13	
PVD	1	1	0	
**Infection**				
EBV high risk	15	9	6	0.584^a^
CMV high risk	30	18	12	0.550^a^
**Type of graft**				
Bilateral	102	64	38	0.236^a^
Single	30	16	14	
**Ischemic times (minutes)**				
Bilateral	313.3 ± 190.1	308.2 ± 188.0	321.7 ± 195.8	0.731^c^
Single	241.4 ± 53.1	239.6 ± 56.1	243.4 ± 51.5	0.849^c^
**AR**				
Total number	31	23	8	
Mean time onset (months)	33.4 ± 38	26.0 ± 37	55.5 ± 32	0.056^c^
**BOS**				
Total number	43	26	17	
Mean time onset (months)	44.2 ± 28	40.6 ± 24	45.4 ± 31	0.600^c^
**Donors**				
Total number	131	73	58	
**Gender**				
Male	58	34	24	0.338^a^
Female	73	39	34	
**Donor age (years)**				
Mean age	45 ± 14	46 ± 13	45 ± 15	0.797^c^
>60	14	8	6	0.571^a^
**Smoking**				
Yes	45	29	16	0.102^a^
No	86	44	42	
**Donor type**				
HB	105	63	42	0.040^a^
Non HB	26	10	16	

*COPD, chronic obstructive pulmonary disease; CF, cystic fibrosis; ILD, interstitial lung disease; PVD, pulmonary vascular disease; EBV, Epstein-Barr virus; CMV, cytomegalovirus; AR, acute rejection; BOS, bronchiolitis obliterans syndrome; HB, heart beating; NHB: non-heart beating*.

a *Fisher's exact test (one-sided) for categorical variables*.

b *χ2 test*.

c *One-way ANOVA for continuous variables*.

The selected C3 rs2230199 SNP, defining whether C3S or C3F is produced, was imputed (info score 1,000) and subsequently subjected to stringent quality control steps, as described in section Patients and Methods (30). A total of 543,637 SNPs and 132 patient and 131 donor samples passed quality control steps.

Of the 132 patients, 64 were transplanted because of chronic obstructive pulmonary disease, 36 because of cystic fibrosis, 31 due to interstitial lung disease, whereas, one patient suffered from pulmonary vascular disease. During the follow-up period, 31 patients presented one or more AR episodes, 43 patients developed BOS, and RAS was not observed. No patient presented pretransplant anti-HLA antibodies, as described previously (31).

**Figure 1:**
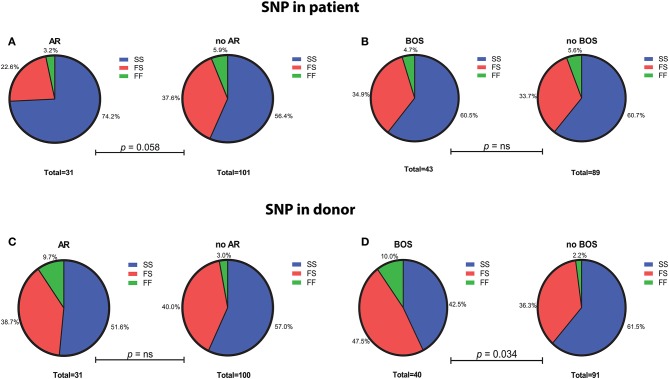
Genotype distribution of the C3 SNP R102G (rs2230199) in LTx patients and donors. **(A)** LTx patients with AR are more often carriers of homozygous slow (SS) variant of the C3 SNP (*p* = 0.058). No significant differences in genotype distribution are found between patients that developed BOS and those without **(B)**. **(C)** Genotype distribution in donors is similar between patients with and without AR, while LTx recipients that developed BOS are more frequently transplanted with donors that carried at least one F allele (*p* = 0.034) **(D)**. Differences in genotype distribution were analyzed with Pearson χ^2^.

SNP distribution in patients passed quality control is as follows: SS 80 (60.6%), SF 45 (34.1%), FF 7 (5.3%), and in donors: SS 73 (55.7%), SF 52 (39.7%), and FF 6 (4.6%) (Figure 1). Normal SNP distribution in the European Caucasian population is: SS 61.4, SF 33, and FF 5.6% (reference population 1,000 Genomes Project Phase 3).

### Presence of C3 Allotype in BALF of LTx Patients

BALF was collected post-transplantation at time of suspected complication (range 4–28 months ±8). None of these patients had developed AR or BOS yet at those time points. To analyze the presence of C3 phenotype in lavage, samples from whom both patient and donor DNA was available were selected (*n* = 6).

**Figure 2:**
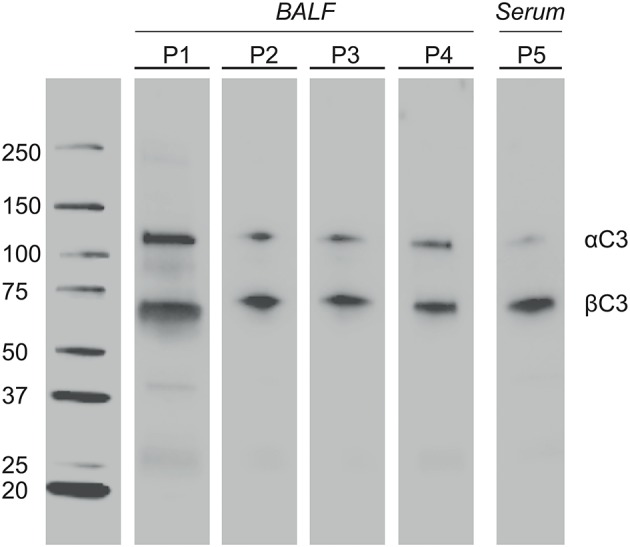
Complement C3 is present in BALF of LTx patients. One-time collected BALF samples of 4 LTx patients (range 4–28 months ±8 post transplantation) were diluted 5x in sample buffer. Samples were heated to 100°C, resolved by SDS-PAGE 4-20% kDa, and transferred to nitrocellulose. Western blotting was performed with a polyclonal antibody to human C3 protein. Lane 1-4: BALF of 4 different LTx patients. Lane 5 (positive control): Serum of a LTx patient (800x dilution). The α′ chain of C3b (110 kD) and iC3b (68 kD) were detected by their molecular weight.

**Figure 3:**
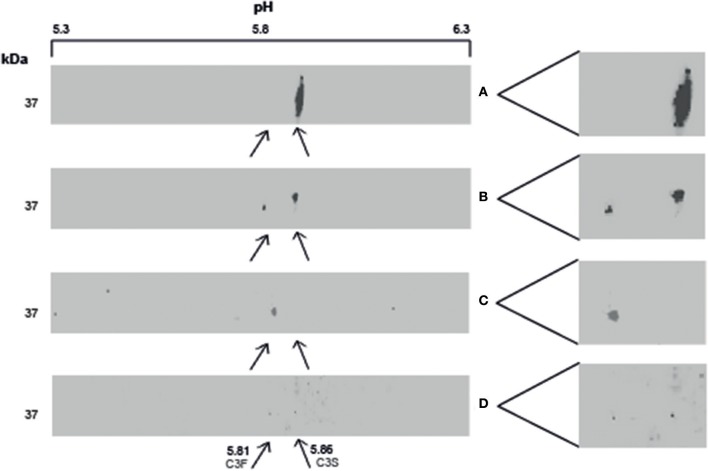
Local production of complement C3 in LTx recipients. 2–3 μg protein in lavage is separated with IEF and SDS-PAGE, followed by western blotting. C3 is specifically detected using a recombinant anti-human C3 antibody. **(A)** (control): BALF of a homozygous C3S LTx patient, transplanted with lungs of a homozygous C3S donor, contains the slow variant of rs2230199 (pI 5.86) at the expected weight of C3d (37 kD). **(B)** (control): lavage of a LTx recipient carrying the heterozygous C3 SNP. **(C)**: BALF of a homozygous C3S LTx patient, transplanted with a homozygous C3F donor, contains the fast C3 variant of rs2230199 (pI 5.81). **(D)** (control): both C3 allotypes were found in a lavage of a C3S patient transplanted with a C3F donor mixed with BALF of a C3S patient transplanted with a C3S donor (1:1).

BALF of 4 patients and serum of 1 patient were separated by SDS-PAGE and Western Blotting. We were able to show for the first time uncleaved C3 in lavage of LTx recipients (Figure 2). C3 levels in BALF are estimated to be 200–400 lower as in serum (calculated by relative band intensities). To demonstrate the production of C3 by donor lungs, proteins in BALF were first separated according to electric point and second to molecular mass. We succeeded to distinguish C3S from C3F on an IPG strip pH 5.3–6.3, visualized by staining C3d using a recombinant anti human C3 antibody (Figures 3A,B). Lavage of a homozygous C3S patient transplanted with a homozygous C3F donor contained the C3F allotype (pI 5.81, 37 kDa), showing the ability of donor lung cells to produce complement C3 (Figure 3C). To confirm these results, lavage of this patient was mixed with lavage of a homozygous C3S patient transplanted with a homozygous C3S donor. Both C3 allotypes could be detected (Figure 3D).

### rs2230199 Influences Clinical Outcome After Lung Transplantation

The C3 rs2230199 SNP configuration from patients as well as donors was tested for association with AR and BOS.

**Figure 4:**
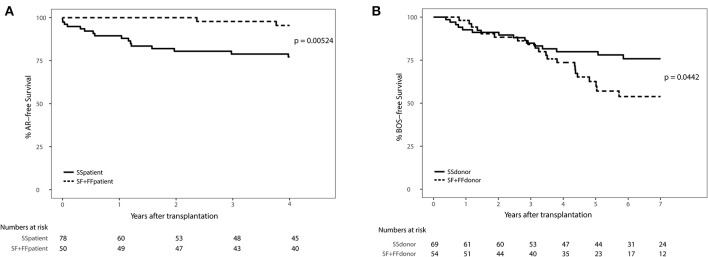
Death-censored AR-free survival **(A)** and BOS-free survival **(B)** in LTx patients stratified per rs2230199 genotype. **(A)** Kaplan-Meier analysis on AR incidence after LTx. A total of 132 patients who passed quality control (QC) were stratified according to rs2230199 genotype, either as homozygous SS, or as carrying a F allele (SF and FF). Patients who deceased within the first 72 h after transplantation were excluded from analysis. Patients genotyped as homozygous SS (solid line) present a lower AR-free survival rate compared to patients genotyped with a F allele (dashed line), *p* = 0.005. Lower table represents numbers at risk. **(B)** Kaplan-Meier analysis on BOS incidence after LTx. Patients who received a graft genotyped FF or FS for this specific SNP have a lower BOS-free survival rate measured over the first 84 months post transplantation (*p* = 0.044). Patients who had deceased within the first 6 months after transplantation or from whom SNP analysis did not pass QC were excluded, resulting in the inclusion of 122 patients. Lower table represents numbers at risk. Log-rank test was used in both analyses.

We excluded patients that deceased within 72 h after transplantation (AR development), resulting in the inclusion of 132 patients (Supplementary Figure 1). We observed a significant association between AR and the SNP configuration of rs2230199 in patients, but not in donors. Patients carrying the homozygous common variant (C3S) present AR episodes more frequently (*p* = 0.005) at 4-years follow-up (Figure 4A) than patients carrying one or two F alleles. We chose a cut-off of 4 years because at that time point most patients (>70%) had already developed AR. This association was proven in a multivariable cox-proportional-hazards model that contains known risk factors for AR development in the intermediate period after LTx (*p* = 0.015, HR 6.2, 95% CI 1.4–27.1) (Table 2) (32). Regarding freedom of chronic rejection, we analyzed survival in LTx patients stratified per donor genotype, with at least 10 subjects remaining at risk in each group. To avoid the bias of postoperative complications in the diagnosis of BOS, patients who died within the first 6 months after transplantation were excluded, resulting in the inclusion of 122 patients (Supplementary Figure 1). The exclusion criteria do not have any effect on the model output (data not shown). We found a significant correlation between patients receiving a graft with at least one F allele and the development of BOS during 7-years follow-up (*p* = 0.044), irrespective of patients' genotype (Figure 4B). This was confirmed in a multivariate analysis using a cox-proportional-hazards model for BOS development, including known donor and patient risk factors for BOS development, and designated the risk variant of rs2230199 as a significant predictor for the development of BOS after LTx (*p* = 0.039, HR 1.4, 95% CI 1.0–2.0) (Table 2).

**Table 2:** Multivariate analysis on AR and BOS incidence in patients treated with lung transplantation.

**AR incidence (*t* = 4jr)**	**Hazard ratio (95% CI)**	***p*-value**
C3S configuration (patients)	6.2 (1.4–27.1)	0.015
CMV reactivation	1.1 (0.4–3.4)	0.834
Year of transplantation	1.0 (1.0–1.0)	0.110
**BOS incidence (*****t*** **= 7jr)**	**Hazard ratio (95% CI)**	***p*****-value**
C3F configuration (donor lungs)	2.1 (1.1–4.3)	0.035
Donor age (≥60)	5.7 (2.1–16.0)	0.001
Donor smoking state	1.0 (0.5–2.1)	0.917
CMV reactivation	0.9 (0.4–2.2)	0.820
EBV reactivation	1.5 (0.6–3.9)	0.436
Recipient age (≥60)	2.5 (1.1–5.9)	0.037
Episode of acute rejection	1.4 (0.7–2.9)	0.343

*AR, acute rejection; BOS, bronchiolitis obliterans syndrome; CMV, cytomegalovirus; EBV, Eppstein-Barr virus; CI, confidence interval*.

## Discussion

In this study, we investigated the relation between clinical parameters after LTx and SNP rs2230199 in complement C3 analyzed in both donors and patients. This study shows that LTx patients genotyped as homozygous C3S have a significant lower AR-free survival and that recipients of donor lungs genotyped with at least one F allele present a lower BOS-free survival. Furthermore, we are the first to show local production of complement C3 by donor lungs which may functionally explain why the donor C3 genotype matters.

To the best of our knowledge, we were the first to show uncleaved C3 in lavage of LTx patients 4–28 months post transplantation, although presence of other complement (activation) components was already demonstrated in lavage of lung transplant recipients (7, 33).

The role of this C3 allotype has frequently been studied in solid organ transplantation. Unfortunately, the results of these studies do not provide a clear picture of the allotype configuration associated with a risk for graft dysfunction, graft survival and rejection episodes (14–19). Despite the importance of the complement system in the development of AR—especially in the early transplantation period—and increased levels of complement factors like C3 and C5a that were found at time of AR (34), no association with AR was found in these studies.

Several studies prove the importance of local complement activation during allograft rejection (35), regardless of systemically produced C3. Tang et al. measured renal C3 production in kidney transplant recipients using a mouse antibody against another polymorphism in C3 closely related to SNP rs2230199 (HAV 4–1),−98% of C3S carriers are HAV 4–1 negative, and 90% of C3F carriers are HAV 4–1 positive–and estimated the donor renal contribution to the total circulating C3 around 4.5% that increases to as much as 16% during rejection episodes (10). The authors found renal donor C3 production 3 to 13 months after transplantation, comparable to our finding that donor C3 is present in lavage at least from 4 to 28 months post transplantation. We hypothesize that in stable transplant patients (as is the case in our cohort at time of BALF collection) ratio donor/recipient C3 is >1, while the ratio decreases in unstable patients due to lung damage and subsequently leaking of recipient complement components into the lung fluid.

Previously, only one paper discriminated C3S and C3F based on their different pI (12). A more recent paper determined C3 polymorphism by capillary electrophoresis, and measured C3c concentration in serum of healthy blood donors according to their C3 phenotype. The authors found significantly lower concentrations for C3F homozygous donors compared to the others (36).

Discrimination of C3S and C3F by their very small difference in pI is technically challenging. Electrophoresis of lavage with a criterion IEF precast gel, pH 5–8 failed due to the low C3 concentration in lavage. Also depletion of albumin and IgG followed by C3 purification with streptavidin magnetic beads was not appropriate as the low pH of the elution buffer (pH 2.9) caused C3 detection problems. In addition, the pH range and the gel length limited a good discrimination between C3S and C3F. Therefore, we chose to separate acetone precipitated C3 first by pI on a 7 cm IPG strip with a narrow pH range (5.3–6.3) and second by molecular mass.

In our cohort most patients develop AR in the early and intermediate phase after LTx. The association between patient SNP rs2230199 and AR after 4-years follow-up reported in our study could be explained by the contribution of the recipient C3 source to AR development. As activation of the classic pathway results in T cell recruitment to the graft, local production of C3 by recipient cells could be involved (37). On the other hand, patients transplanted with a lung genotyped with at least one F allele, have a lower BOS-free survival rate. To our knowledge, we are the first to show this association in LTx recipients. It has been suggested that the SNP rs2230199 affects functional activity. Heurich et al. demonstrate with pure protein that C3F in combination with 2 other SNPs in factor B and factor H promotes alternative pathway activity, whereas C3S results in a less active complement system which may increasing the susceptibility to infection (38). This difference in hemolytic activity between C3 allotypes could explain why C3S in patients is related to AR since a main risk factor for AR development is (recurrent) infections, while the higher BOS incidence observed in patients receiving a C3F donor lung graft is probably due to their increased hemolytic complement activity. Importantly, the role of this particular SNP in the C3 gene in AR and BOS warrants further investigation to better understand the mechanisms behind our findings.

AR and BOS were diagnosed according to international guidelines, but not proven by transbronchial lung biopsies since surveillance bronchoscopy and BALF analyses are not performed in our center, which is a limitation of this study. Although in previous studies concerning the association of SNP rs2230199 in the C3 gene with graft outcome after transplantation (donor-recipient) pairs with different combinations of donor and recipient C3 alleles were related to graft outcome after transplantation, we have not included these analyses due to the low frequency of homozygous F donors and recipients. Furthermore, because these data are inconclusive, and a limited number of samples were analyzed in this study, our findings need to be replicated, and validated in a larger cohort. Additional studies are also required to confirm the C3 IEF analysis which was performed only in a few BALF samples due to sample availability. In addition, the methodology applied did not allow quantification of C3S and C3F concentrations in lavage, which seems to be relevant (36), precluding separation between locally and systemically produced C3. Also the ratio between donor and recipient C3 could be of interest, as well as the concentration of complement activation products. While it is interesting to investigate complement involvement in the local C3 production by donor lung cells, it is beyond the scope of this study to explore the mechanisms behind our results. Lastly, we do not know the influence of several demographic characteristics like type of immunosuppressive drugs, gender, and age on complement activity (CH50), and complement C3 levels.

In the last few years, targeted SNP analyses focused on the role of the complement system in solid organ transplantation have increased (4, 39, 40). Our group previously reported a genetic promoter polymorphism in several complement regulatory proteins to be associated with chronic rejection after LTx (41), and with kidney transplant outcome (42). These findings indicate the importance of this emerging field.

To summarize, we showed local C3 production by donor grafts in recipients treated with LTx. Furthermore, our data indicate an effect of a C3 polymorphism on both short- and long-term outcome after LTx. Importantly, larger study cohorts are needed to confirm our data. Nevertheless, our results could be used for a better prediction of patients at risk to develop rejection after LTx.

## Data Availability Statement

The datasets generated for this study can be found in EGA at https://ega-archive.org, reference number EGAS00001003843.

## Ethics Statement

Written informed consent was obtained from all study participants and this study was approved by the medical ethical committee of the University Medical Center Utrecht (METC 06–144).

## Author Contributions

TK-H, KB, JS, and OR performed the research. TK-H, JS, EG,OR, and HO participated in data analysis. EG and E-JO contributed patient material. EG and HO participated in research design. TK-H, KB, EG, JS, and HO wrote the paper. All authors provided final approval of the version to be published.

### Conflict of Interest

The authors declare that the research was conducted in the absence of any commercial or financial relationships that could be construed as a potential conflict of interest.

## Supplementary Material

Supplementary Figure 1 Flowchart of included samples for SNP analysis and reasons for excluding samples. In total 144 transplantations were included. Samples were genotyped for SNP rs2230199 and applied to stringent QC procedures as described in section Patients and Methods. 12 LTx recipients and 13 donors did not passed QC for SNP analysis. For survival analyses patients that died within 72 h after transplantation (AR) or within 6 months (BOS) were also excluded. SNP, single nucleotide polymorphism; QC, Quality Control; LTx, lung transplantation; AR, acute rejection; BOS, bronchiolitis obliterans syndrome; KM, Kaplan Meyer.

## Abbreviations

LTx: lung transplantation
AR: acute rejection
CLAD: chronic lung allograft dysfunction
RAS: restrictive allograft syndrome
BOS: bronchiolitis obliterans syndrome
BALF: bronchoalveolar lavage fluid
C3S: homozygous C3 slow phenotype
C3F: homozygous C3 fast phenotype
SNP: single nucleotide polymorphism
pI: isoelectric point
CMV: cytomegalovirus
IEF: isoelectric focusing.
