# Supplementary figures and images for: A Single Nucleotide C3 Polymorphism Associates With Clinical Outcome After Lung Transplantation
Source: Front Immunol. 2019 Sep 26;10:2245. doi: 10.3389/fimmu.2019.02245 (PMC6775212; doi:10.3389/fimmu.2019.02245)

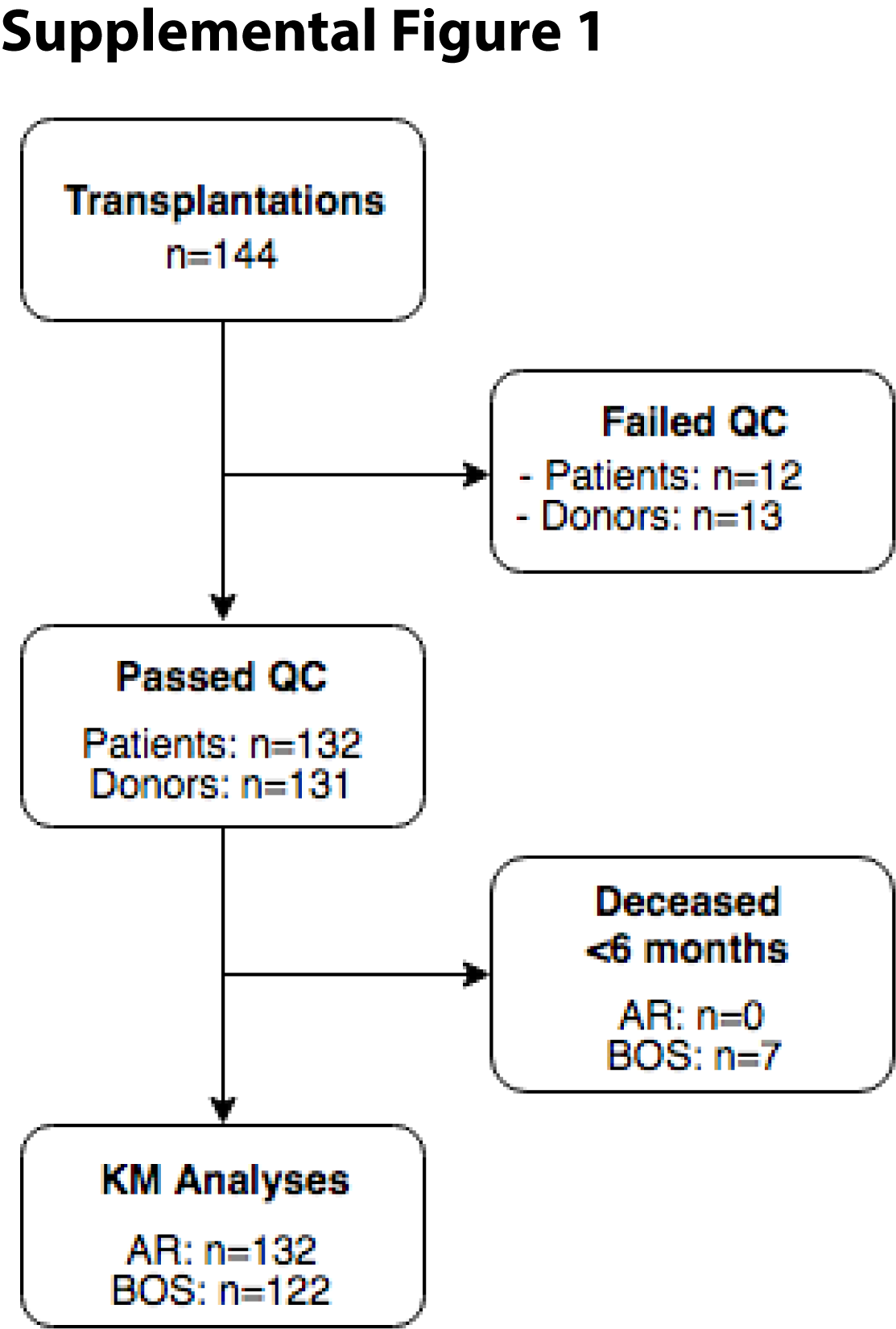

Supplement: Supplementary Figure 1 — Flowchart of included samples for SNP analysis and reasons for excluding samples. In total 144 transplantations were included. Samples were genotyped for SNP rs2230199 and applied to stringent QC procedures as described in section Patients and Methods. 12 LTx recipients and 13 donors did not passed QC for SNP analysis. For survival analyses patients that died within 72 h after transplantation (AR) or within 6 months (BOS) were also excluded. SNP, single nucleotide polymorphism; QC, Quality Control; LTx, lung transplantation; AR, acute rejection; BOS, bronchiolitis obliterans syndrome; KM, Kaplan Meyer. [file Image_1.TIF]
